# Preparation of Bio-Based Aerogel and Its Adsorption Properties for Organic Dyes

**DOI:** 10.3390/gels8110755

**Published:** 2022-11-21

**Authors:** Penghui Li, Chi Yang, Xuewen Xu, Chen Miao, Tianjiao He, Bo Jiang, Wenjuan Wu

**Affiliations:** 1 Jiangsu Co-Innovation Center of Efficient Processing and Utilization of Forest Resources, Nanjing Forestry University, Nanjing 210037, China; 2 College of Light Industry and Food Engineering, Nanjing Forestry University, Nanjing 210037, China

**Keywords:** aerogel, cellulose, starch, adsorption

## Abstract

The effective utilization of biomass and the purification of dye wastewater are urgent problems. In this study, a biomass aerogel (CaCO_3_@starch/polyacrylamide/TEMPO-oxidized nanocellulose, CaCO_3_@STA/PAM/TOCN) was prepared by combining nanocellulose with starch and introducing calcium carbonate nanoparticles, which exhibited a rich three-dimensional layered porous structure with a very light mass. Starch and nanocellulose can be grafted onto the molecular chain of acrylamide, while calcium carbonate nanopores can make the gel pore size uniform and have excellent swelling properties. Here, various factors affecting the adsorption behavior of this aerogel, such as pH, contact time, ambient temperature, and initial concentration, are investigated. From the kinetic data, it can be obtained that the adsorption process fits well with the pseudo-second-order. The Langmuir isotherm model can fit the equilibrium data well. The thermodynamic data also demonstrated the spontaneous and heat-absorbing properties of anionic and cationic dyes on CaCO_3_@STA/PAM/TOCN aerogels. The adsorption capacity of Congo red (CR) and methylene blue (MB) by CaCO_3_@STA/PAM/TOCN was 277.76 mg/g and 101.01 mg/g, respectively. Therefore, cellulose and starch-based aerogels can be considered promising adsorbents for the treatment of dye wastewater.

## 1. Introduction

With the rapid industrial development of modern society, the issue of environmental pollution (especially water pollution) has become a worldwide hot topic [1]. Water pollution has become an urgent environmental issue, with over 50,000 tons of industrial dyes reportedly being discharged into rivers or the sea every year [2]. The disadvantage of textile dyes in wastewater is that they can reduce water reoxygenation capacity and complicate the steps to treat wastewater [3]. Methods to treat dyes in water pollution have emerged. Currently, there are many effective methods for removing organic dyes from wastewater, such as membrane separation [4], biodegradation [5], catalytic degradation [6], and adsorption [7]. Adsorption methods are widely used because of their ease of operation, low energy consumption, and essentially no secondary contamination. For adsorption, the presence or absence of abundant adsorption sites is the key to evaluating a good adsorbent.

Aerogels are solid materials [8] that replace the liquid or gel phase with a gas in a three-dimensional mesh structure. It has many features ideal for adsorption, such as large internal surface area, wide pore size distribution, rapid recovery from aqueous solutions, and low density, which will be developed to meet various industrial wastewater treatment needs and greatly increase the adsorption capacity of the adsorbent [9]. Compared with traditional aerogels, biomass aerogels also have the advantages of being economical and environmentally friendly, biodegradable, and easily accessible [10]. Currently, classical biomass-based aerogels include lignin-based aerogels [11], chitosan-based aerogels [12], and cellulose-based aerogels [13]. Cellulose (CE) is considered a promising raw material for preparing biomass aerogels due to its unique linear structure and mechanical properties. It is also the world’s most abundant renewable biological resource. In addition, cellulose contains many hydroxyl groups, making it a good adsorbent for organic dyes [14,15]. However, the hydroxyl groups of cellulose may form hydrogen bonds with substances in the wastewater, leading to a decrease in the affinity of the adsorbent. Cellulose-based aerogels need to be modified to enhance the adsorption effect [16]. For TEMPO-oxidized nanocellulose, the particle size distribution is uniform, and the carboxyl group content is high, which enhances the stability of the gel itself. Secondly, drying the gels is essential for the formation of porous structures. Cellulose is a polysaccharide chain that tends to keratinize during drying. Polyhydroxy substances (e.g., starch) prevent synergistic hydrogen bonding of cellulose chains by introducing space-site blocking or electrostatic groups, effectively mitigating keratinization and maintaining our desired porous structure [17]. In the absence of cross-linking agents, starch can form a complete gel network structure. Starch-based aerogels, with advantages such as low density (0.10 g/cm^3^—0.24 g/cm^3^) and low thermal conductivity (0.024 W/m·K—0.043 W/m·K) [18]. Wang et al. [19] prepared aerogels from konjac glucomannan and starch using environmentally friendly sol-gel and freeze-drying methods and showed that starch addition significantly enhanced the mechanical strength of the aerogels. It is worth mentioning that calcium carbonate also has good performance in wettability, hydrophobicity, and particle adhesion, in addition to basic properties such as low cost, non-toxicity, and biocompatibility [20]. In contrast, nano-CaCO_3_ has a small particle size, surface size effect, and macroscopic quantum tunneling effect, with the disadvantage of poor dispersion in water, which is detrimental to the adsorption process [21]. Previously, CaCO_3_ has been applied to the adsorption of Congo red dye [22]. Chong et al. [23] loaded CaCO_3_ onto cellulose aerogels by in-situ precipitation of CaCO_3_, which led to the adsorption of Congo red. The results showed that CaCO_3_ incorporation into the cellulose aerogel significantly enhanced the adsorption capacity of the aerogel for the dye.

In this study, an environmentally friendly multifunctional biomass-based aerogel (CaCO_3_@STA/PAM/TOCN) was prepared for the removal of anionic and cationic dyes using a one-step sol-gel and freeze-drying method using TEMPO-oxidized nanocellulose, tapioca starch, and calcium carbonate nanoparticles. The morphological structure of the prepared aerogel was systematically characterized, and the effects of pH, contact time, temperature, and other variables on the adsorption effect were discussed. Finally, the kinetic, isothermal, and thermodynamic models of the adsorption process were investigated in detail.

## 2. Results and Discussion

### 2.1. Characterization of Aerogels

**Figure 1:**
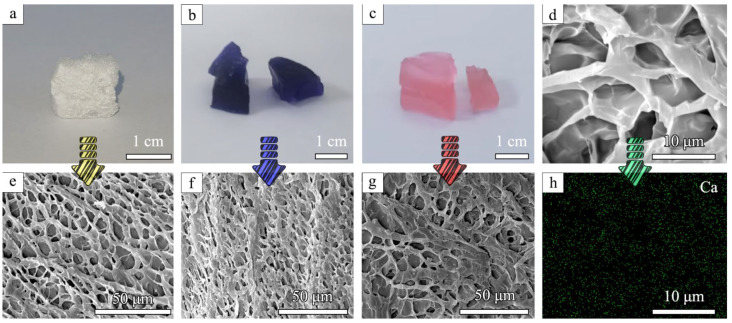
Digital photos of the (**a**) CaCO_3_@STA/PAM/TOCN aerogel; (**b**) CaCO_3_@STA/PAM/TOCN aerogel after adsorption of methylene blue; (**c**) CaCO_3_@STA/PAM/TOCN aerogel after adsorption of Congo red; SEM images of the (**d**,**e**) CaCO_3_@STA/PAM/TOCN aerogel; (**f**) CaCO_3_@STA/PAM/TOCN aerogel after adsorption of methylene blue; (**g**) CaCO_3_@STA/PAM/TOCN aerogel after adsorption of Congo red; (**h**) EDX mapping images of the CaCO_3_@STA/PAM/TOCN.

**Scheme 1:**
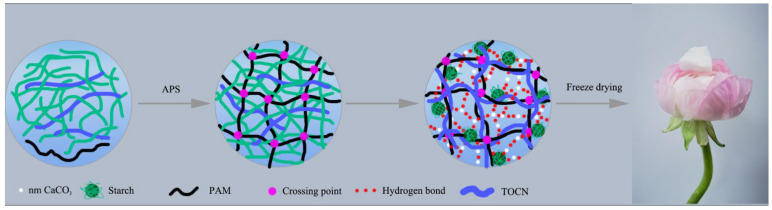
Schematic illustration of the preparation of CaCO_3_@STA/PAM/TOCN aerogel.

The surface morphology and internal structure of the prepared aerogels were observed by SEM. CaCO_3_@STA/PAM/TOCN showed a flexible, porous skeleton with a homogeneous channel structure, which facilitated ion diffusion and liquid permeation during dye adsorption (Figure 1d–g). In contrast, pure CaCO_3_-doped cellulose presents a bundle structure with an unsmooth surface [23]. The homogeneous pore distribution of the gel is due to its ease of forming hydrogen bonds between the molecular chains of starch, nanocellulose, and polyacrylamide. This makes the nano-calcium carbonate a cohesive center, thus making the molecular chains more tightly based [24]. It is observed from Scheme 1 that the synthesized aerogel is light in mass, and a piece of CaCO_3_@STA/PAM/TOCN with dimensions of 1.5 × 1.5 × 0.5 cm is easily supported by several petals due to its light density (42 mg/cm^3^). Figure 1a shows the morphology of the prepared aerogel, and Figure 1b,c shows the morphology of the aerogel adsorbed with methylene blue and Congo red.

The elemental mapping technique was used to further investigate the structural element distribution, as shown in Figure 1h. The elemental mapping results showed that Ca was uniformly distributed on the aerogel surface. CaCO_3_ particles were uniformly precipitated around, and the starch became active after cooking and complete pasting, allowing bonding [25]. This uniform distribution of inorganic nanoparticles in the aerogel adsorbent network may improve dye adsorption efficiency [26].

**Figure 2:**
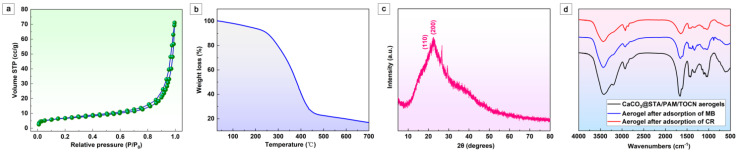
(**a**) Nitrogen adsorption–desorption isotherms of CaCO_3_@STA/PAM/TOCN aerogels; (**b**) TGA results of CaCO_3_@STA/PAM/TOCN aerogels; (**c**) XRD patterns of CaCO_3_@STA/PAM/TOCN aerogels; (**d**) FITR of CaCO_3_@STA/PAM/TOCN aerogels.

Figure 2a shows the adsorption/desorption isotherm curves of the aerogel obtained for N_2_ at 77 K. The BET method shows a specific surface area of 39.23 m^2^/g. The average pore size is 9.87 nm. The lower specific surface area of CaCO_3_@STA/PAM/TOCN is due to fewer micropores/mesopores. The BET area measurement matches well with the morphology observed by SEM characterization [27]. The thermal behavior of CaCO_3_@STA/PAM/TOCN aerogels was analyzed using TGA, and the curves were plotted as a function of temperature, as shown in Figure 2b. The thermal decomposition of the biomass aerogel is divided into three stages in Figure 2b. In the first stage, a small weight loss of the aerogel is observed below 230 °C, corresponding to the evaporation of water. As previously reported, a severe mass reduction is observed in the phase around 270–420 °C, which may be related to the depolymerization and decomposition of glucose units in cellulose [28]. The mass loss of 9.28% in the final stage (400–600 °C) is attributed to the decomposition of the aerogel cross-linked structure and further conversion to CO_2_ and H_2_O [29]. The XRD patterns obtained for CaCO_3_@STA/PAM/TOCN are shown in Figure 2c. CaCO_3_@STA/PAM/TOCN exhibits a typical cellulose II diffraction pattern, where the peaks at 2θ = 20.2° and 22.2° correspond to the (110) and (200) crystal planes, respectively [23]. The XRD patterns obtained for CaCO_3_@STA/PAM/TOCN confirm that the lattice structure is mainly the calcite phase, in agreement with the SEM observations (Figure 1e). Owing to starch pasting, strong diffraction peaks were not observed in the prepared aerogels. The FTIR spectra before and after aerogel adsorption are shown in Figure 2d. After cellulose aerogel, three strong peaks appear at 3337 cm^−1^, 1450 cm^−1^, and 1154 cm^−1^, attributed to the O-H, H-C-H, and C-O-C bands in the cellulose group, respectively. The peak at 1618 cm^−1^ was attributed to the water absorbed by the cellulose. As seen in Figure 2d, a slight blue shift and weakening of the broadband of the OH group of the hydrogen bond occur. It is possible that the hydrogel bonds are formed between functional groups and OH groups in MB or CR. In addition, the peak of the vibrational mode belonging to the methylene group at 2931 cm^−1^ is shifted to 2923 cm^−1^, indicating that hydrophobic interactions are involved in addition to the electrostatic interactions between the dye and the aerogel. Thus, the adsorption mechanism of CaCO_3_@STA/PAM/TOCN on CR is mainly electrostatic attraction, hydrogen bonding and there is a transfer of electrons in the process, and the adsorption mechanism on MB is hydrogen bonding adsorption and electrostatic attraction.

### 2.2. Study of the Adsorption Properties of MB and CR

#### 2.2.1. Effect of Solution pH

**Figure 3:**
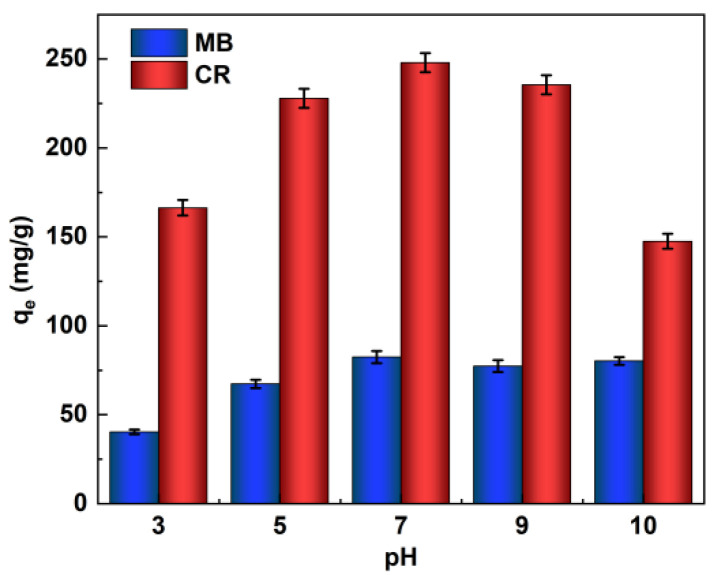
Effects of initial pH on the MB and CR adsorption capacity (adsorption time t = 6 h, ambient temperature T = 20 °C).

The pH of the initial solution can significantly affect the adsorption process of dyes because the surface charge of the adsorbent and the degree of ionization of the dye, and even the structure of the dye molecules, can change with pH. In order to make the CaCO_3_@STA/PAM/TOCN adsorbent achieve the best adsorption effect (q_e_: adsorption at equilibrium, mg/g) on MB and CR in a suitable acidic and alkaline environment, we set different gradients of pH (3–10) to determine the best conditions for the experiment, as shown in Figure 3. Both the concentrated acidic and basic environments affect the conjugated structure of dye molecules.

The adsorption capacity of the MB and CR reached the maximum adsorption capacity at neutral or weakly basic, and the adsorption capacity increased with increasing pH (pH 3–7), and both dyes were comparable at a pH of 7–9. For MB, in an acid solution, the presence of large amounts of hydrogen ions around the adsorption site hinders the approach of MB, and the protonated amino and hydroxyl groups on the sugar molecule chains generate electrostatic repulsion, leading to a decrease in the adsorption capacity. In alkaline solutions, the aerogel is deprotonated, and the electrostatic attraction between the aerogel and the cationic MB leads to a higher adsorption capacity [30]. For the CR dye, the adsorption mechanism is slightly different. Under alkaline conditions, the electrostatic repulsion decreased with increasing pH, increasing the adsorption capacity; when the pH continued to increase, the gravitational force between the aerogel and CR also decreased, and the adsorption capacity tended to decrease. In acid solutions, electrostatic repulsion between protonated aerogels and cationic CR molecules leads to a decrease in adsorption capacity as the pH decreases due to the presence of nitrogen atoms and sulfonate groups in the acid solution [31,32]. Thus, the adsorption of cationic and anionic dyes was strongly influenced by the change in pH.

#### 2.2.2. Effect of Contact Time

**Figure 4:**
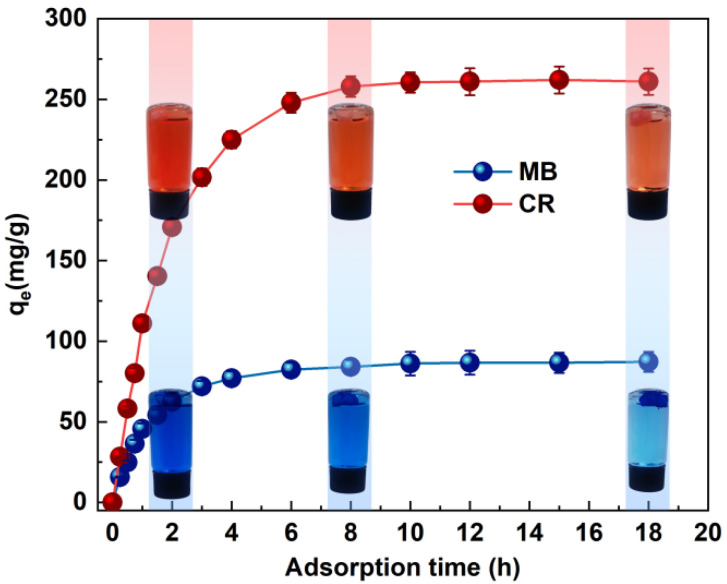
Effects of the adsorption time on the MB and CR adsorption capacity (pH value pH = 7, ambient temperature T = 20 °C).

Contact time is one of the important factors in determining dye removal. The effect of MB and CR dye adsorption is shown in Figure 4. The adsorption trends of CR and MB are approximately the same. The trend of adsorption was roughly divided into three stages, i.e., the initial rapid adsorption stage, followed by a gradual decrease in the adsorption rate, and finally reaching the equilibrium state. The adsorption of MB and CR dyes gradually reached the optimum effect within 6–8 h. The contact time and percentage removal efficiency increased from 15 min (17.24%) to 6 h (94.56%) for MB and CR, respectively, and the contact time and removal efficiency percentages increased from 15 min (10.76%) to 6 h (95.13%), respectively. In the first region, the adsorbent surface was rich in active sites and dyes aggregated on the monolayer of the adsorbent surface. As the adsorption time increased, both pores and active sites were occupied [33]. In the second time period, the saturation of the active sites on the outer surface is observed, and the adsorption capacity can only increase slowly. In the last region, dye molecules penetrate into the adsorbent pores, and the adsorption capacity remains essentially unchanged due to the small repulsive forces between the free dye molecules in solution and those adsorbed on the adsorbent surface, which may be related to the resistance to dye adsorption [34].

#### 2.2.3. Influence of Ambient Temperature

**Figure 5:**
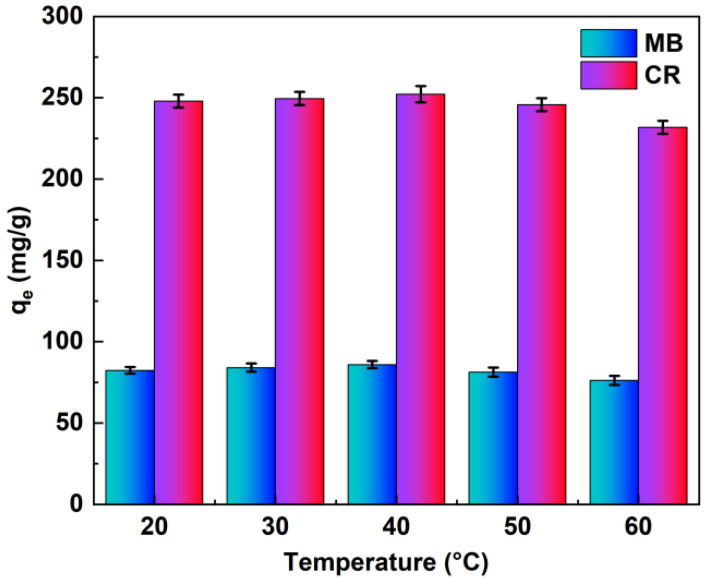
Effects of ambient temperature on the MB and CR adsorption capacity (pH value pH = 7, adsorption time t = 6 h).

To investigate the effect of ambient heat on the dye adsorption process, we conducted experiments at different temperatures (20 °C, 30 °C, 40 °C, 50 °C, and 60 °C), as shown in Figure 5. In the range of 20–40 °C, the adsorption capacity was essentially flat, and 30–40 °C was the optimum adsorption temperature, at 30–60 °C indicating a slight decrease in adsorption capacity with increasing temperature, which is unfavorable for adsorption. The possible reason for this is that the dye adsorption reaction process is exothermic, and high temperatures contribute to the inability of the reaction to proceed [35]. It can also be seen from Figure 5 that the adsorbent performed satisfactorily at all the temperatures studied, and it shows that temperature does not have much influence on the adsorption process [36].

#### 2.2.4. Adsorption Kinetics, Isotherms, and Thermodynamics

**Figure 6:**
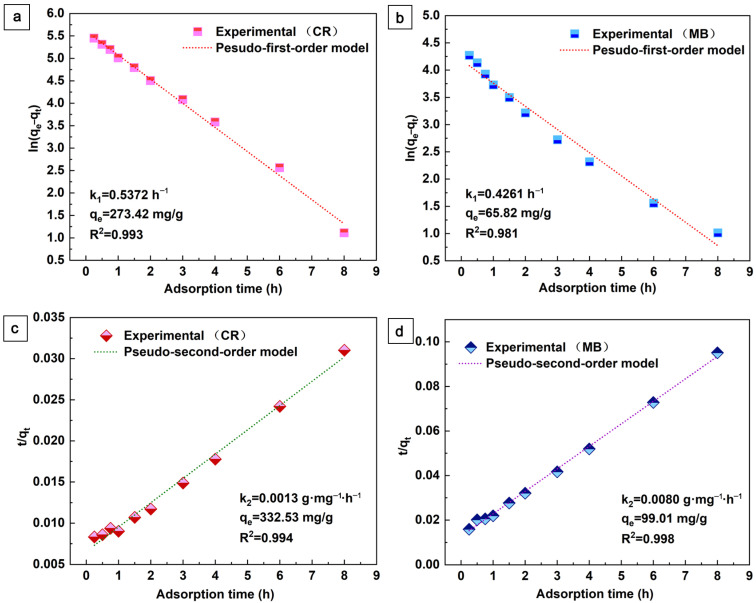
Pseudo-first-order and pseudo-second-order kinetic models for the adsorption of CR (**a**,**c**) and MB (**b**,**d**) on the aerogel.

To evaluate the kinetic mechanism of MB and CR adsorption, Figure 6 shows the fitted data through pseudo-first-order and pseudo-second-order kinetic models [37], Equations (1) and (2).

(1) $$q_{t}=q_{e}\left(1-e^{-k_{1}t}\right)$$

(2) $$q_{t}=q_{e}\left(1-\frac{1}{1+k_{2}q_{e}t}\right)$$

Here, k_1_ (h^−1^) and k_2_ (g·mg^−1^·h^−1^) are the rate constants. The fitted kinetic parameters are shown in Figure 6. As can be seen in Figure 6, the pseudo-second-order kinetics of the fit coefficients (R^2^ > 0.994) for CR and MB are better than the proposed first-order kinetics. This indicates that the adsorption is mainly controlled by the multiplicative decision step. In addition, the pseudo-second-order model considers the chemosynthetic interactions between the adsorbent and the adsorbate in chemisorption. The lower rate constant (k_2_) indicates that the adsorption rate decreases with increasing contact time, which may be related to the reduction of active sites during the adsorption process [37]. In this work, the Lagrangian pseudo-second-order model is more consistent with the adsorption process of aerogels, indicating adsorption with predominantly shared or exchanged electrons between the adsorbent and the adsorbate, which is consistent with most reports on aerogel adsorbent.

In addition, the adsorption behavior of aerogels was described using the intraparticle diffusion model proposed by Weber and Morris:

(3) $$q_{t}=K_{id}t^{0.5}+C_{i}$$

**Figure 7:**
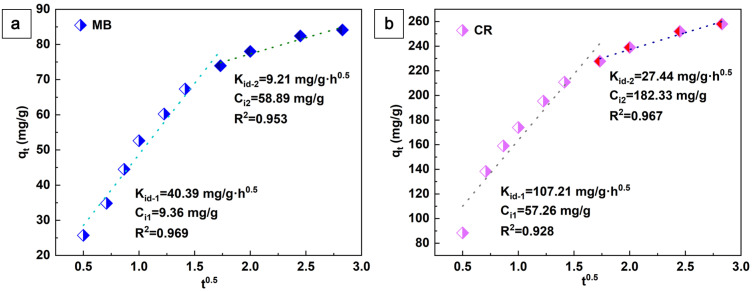
The linear dependence of q_t_ on t^0.5^ based on the intra-particle diffusion, MB (**a**) and CR (**b**).

Here, *K_id_* (mg·g^−1^·h^0.5^) and *C_i_* (mg·g^−1^) denote the intraparticle diffusion rate constant and the parameter associated with the boundary layer thickness, respectively. The values of these two parameters can be obtained by plotting *q_t_* versus *t*^0.5^, as shown in Figure 7. This plot is divided into two separate regions rather than a single linear plot through the origin. This indicates that the adsorption process of the aerogel on CR and MB is a two-step process. In addition to the effect of intraparticle diffusion, boundary layer diffusion also affects the adsorption of dyes. In the second stage, the rate of intraparticle diffusion decreases accordingly due to the simultaneous decrease in dye concentration and adsorption sites [38]. The adsorption of MB and CR is a multi-stage process that includes the surface adsorption of cellulose-based aerogels and the porous structure [39].

**Figure 8:**
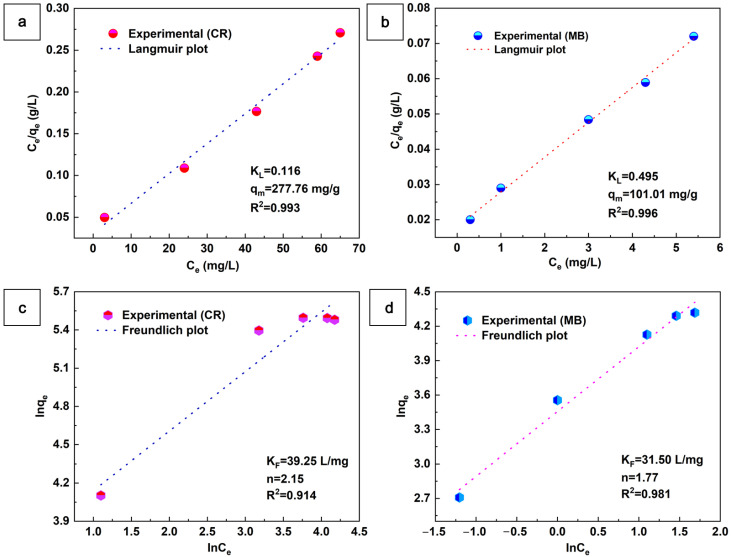
Langmuir and Freundlich isotherms for the adsorption of CR (**a**,**c**) and MB (**b**,**d**) on the aerogel (m = 10 mg, V = 50 mL, t = 6 h).

The adsorption behavior of the aerogel adsorbents was investigated using the Langmuir and Freundlich models, as shown in Figure 8. The Langmuir model assumes that adsorption is limited to a monolayer on a homogeneous surface with identical adsorption sites and no chemical reactions, while the Freundlich model assumes that adsorption on multilayer adsorption on heterogeneous surfaces that allow adsorbed molecules to interact. The equations of the Langmuir and Freundlich models can be expressed as follows:

(4) $$\frac{C_{e}}{q_{e}}=\frac{C_{e}}{q_{m}}+\frac{1}{q_{m}K_{L}}$$

(5) $$Inq_{e}=InK_{F}+\frac{1}{n}InC_{e}$$

K_L_ (L/mg) is the Langmuir constant, and q_m_ (mg/g) is the maximum adsorption capacity. K_L_ and q_m_ can be determined by plotting C_e_/q_e_ versus C_e_ linearly. K_F_ ((mg/g)·(L/mg)^1/n^) is the Freundlich constant associated with the adsorption capacity, and 1/n is the adsorption strength. K_F_ and 1/n can be determined by plotting the intercept and slope of the linear graph of Inq_e_ versus lnC_e_. All of the above parameters can be found in Figure 8. As seen in Figure 8, the CR and MB values of the Langmuir correlation (R^2^) are higher than those of the Förster isotherm model. This indicates that the Langmuir isotherm model dominates the aerogel adsorption CR and MB. Moreover, the maximum adsorption capacity (q_m_) of CaCO_3_@STA/PAM/TOCN aerogel for the adsorption of CR and MB was 277.76 and 101.01 mg/g, respectively. The adsorption capacity of CaCO_3_@STA/PAM/TOCN for adsorption of the anionic dyes CR was greater than that of the cationic dyes MB [40].

The adsorption of the MB and CR dyes on CaCO_3_@STA/PAM/TOCN was carried out at different temperatures of 293, 303, and 313 K, respectively. Different thermodynamic parameters of adsorption such as Gibbs free energy change (ΔG^0^ (kJ·mol^−1^)), enthalpy change (ΔH^0^ (kJ·mol^−1^)), and entropy change (ΔS^0^ (J·mol^−1^·K^−1^)) were calculated using the following equations [33]:

(6) $$Ink=\frac{∆S^{0}}{R}-\frac{∆H^{0}}{RT}$$

(7) $$k=\frac{q_{e}}{C_{e}}$$

(8) $$∆G^{0}=-RTInk$$

where *k* is the rate constant. *R* is the ideal gas constant (8.314 × 10^−3^ J·mol^−1^·K^−1^). *T* is the specified temperature (K). The values of Δ*H*^0^ and Δ*S^0^* are calculated based on the slope and intercept of Ink versus 1/*T*.

**Figure 9:**
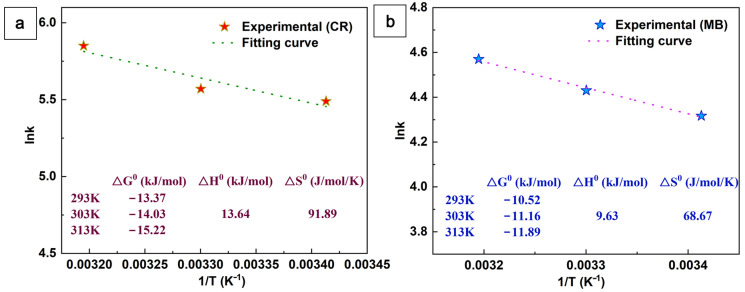
Plots of lnk against 1/T for the adsorption of CR (**a**) and MB (**b**) onto aerogel.

Figure 9 shows the thermodynamic parameters of the CR and MB dye adsorption on CaCO_3_@STA/PAM/TOCN. The positive values of Δ*H^0^* and Δ*S*^0^ indicate that the dye adsorption process CaCO_3_@STA/PAM/TOCN is heat absorbing and is a stochastic increasing process. This heat absorption property indicates a more favorable dye adsorption process at higher temperatures. In addition, the negative value of ΔG^0^ reveals that the dye adsorption process on CaCO_3_@STA/PAM/TOCN is spontaneous.

#### 2.2.5. Recycle Performance

**Figure 10:**
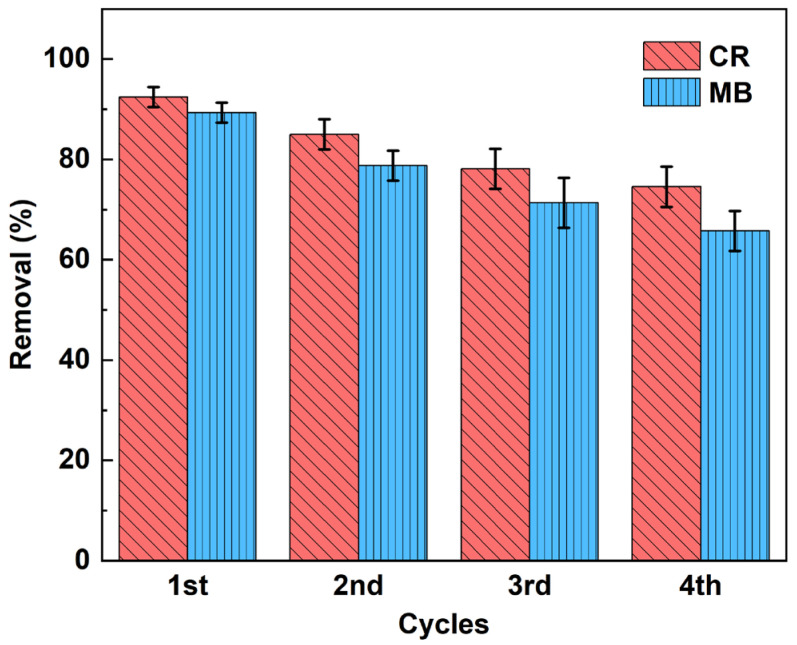
Cyclic adsorption of CaCO_3_@STA/PAM/TOCN aerogels (reusability experiments were performed at pH 7.0 with a dose of 10 mg of CaCO_3_@STA/PAM/TOCN, an elution volume of 25 mL at 20 °C, an initial CR dye concentration of 100 mg/L, an initial MB dye concentration 4 mg/L. After each elution, the aerogels were freeze-dried at −40 °C).

In general, good adsorbents have a high adsorption capacity and a high desorption efficiency, which can significantly reduce the cost of preparing the adsorbent. For the sustainable application of the adsorbent, the reusable performance of the aerogel was investigated, as shown in Figure 10. Twenty-five ml of hydrochloric acid (0.1 mol/L) was chosen as the elution solvent, and the adsorbent was collected after elution (approximately 10 min), rinsed with distilled water, and finally freeze-dried and reused for MB and CR removal [41]. In Figure 10, we found that the macropore desorption was relatively easy, and the adsorption efficiency gradually decreased in each cycle. This decrease may be due to the pore loss of aerogel during the adsorption-desorption cycle with its own degradation and due to the permanent occupation of some adsorption sites by solvent molecules [42]. After four adsorption cycles, the adsorption rate of CR was about 74.56%. The adsorption rate of MB was about 65.75%. The results indicate that CaCO_3_@STA/PAM/TOCN can be repeatedly used as an efficient adsorbent for dye wastewater treatment.

#### 2.2.6. Comparison of Aerogel Adsorption Performance

As shown in Table 1, the CaCO_3_@STA/PAM/TOCN aerogel has a considerable adsorption effect on cationic dyes MB and anionic dyes CR compared to the literature survey of published works. It has abundant functional groups and a rich pore structure with the ability to adsorb and remove both types of dyes of anion and cation from an aqueous solution.

## 3. Conclusions

Cellulose-based biomass aerogel adsorbent materials have received a lot of attention in recent years. Starch and cellulose are the most common polysaccharide materials on earth. They offer many advantages, such as biodegradability, good stability, non-toxicity, environmental friendliness, a wide range of sources, renewability, and low price. The aerogel is a bio-based aerogel prepared by TEMPO-oxidized cellulose nanofibers, starch, calcium carbonate nanoparticles, and acrylamide together. The prepared aerogel has a regular and non-collapsing three-dimensional mesh structure with pores consisting of large pores, which greatly improves its water absorption and water retention and has an excellent ability to adsorb macromolecules. The CaCO_3_@STA/PAM/TOCN aerogel adsorbs the organic dye CR and MB mainly through electrostatic interactions and hydrogen bonding. The calculated adsorption data are consistent with a pseudo-second-order kinetic model and follow the Langmuir adsorption isotherm. The equilibrium adsorption amounts of CR and MB for this aerogel were 277.76 and 101.01 mg/g, respectively, obtained from the Langmuir curve. The aerogel is a green and environmentally friendly material, and the adsorption efficiency of the aerogel is between 60–80% after four cycles. By the considerable adsorption of anionic and cationic types of dyes, we infer that this aerogel is promising. However, the only drawback is that the preparation step of this aerogel is complicated, and future work can choose a simpler approach to prepare it.

## 4. Materials and Methods

### 4.1. Materials

Congo red (CR), methylene blue (MB), acrylamide, N-N’ methylene bisacrylamide, NaOH, and HCl were purchased from Sinopharm Chemical Reagent Co., Ltd., Shanghai, China. Ammonium persulfate was purchased from Aladdin (Shanghai, China). Cassava starch was from Yuanye Biotechnology Co., Ltd., Shanghai, China. TEMPO oxidation of nanocellulose was performed in the lab, with a carboxyl content of 1.85 mmol/g. All the materials were used without purification.

### 4.2. Analytical Measurements of MB and CR Concentration

Maximum absorption wavelength determination: Congo red and methylene blue were accurately weighed for the corresponding organic pollutants, and 100 mg/L of dye solution was accurately configured in volumetric flasks and formulated into different concentration gradients. The absorption or reflectance in the visible range directly affects the perceived color of the chemicals involved. The full spectrum of the two organic pollutant solutions can be scanned separately by a UV spectrophotometer (UV2200, Shanghai Sunyu Hengping Scientific Instruments Co., Ltd., Shanghai, China) to determine the maximum absorption wavelength of the dyes.

Standard curve determination: Different concentration gradients of CR (20 mg/L, 40 mg/L, 60 mg/L, 80 mg/L, and 100 mg/L) and MB (1 mg/L, 2 mg/L, 2.5 mg/L, 3 mg/L, and 4 mg/L) were prepared, and the absorbance at the maximum absorption wavelength of the corresponding organic pollutant solutions were determined at different concentrations. The linear relationship between the absorbance of organic pollutants and the concentration of organic pollutants was fitted, and the merit of the fitted relationship was judged by the correlation coefficient R^2^ (controlled at about 0.9). As a result, the standard curves of the organic pollutant solutions were obtained, and the basic information about the two organic dyes and the relevant parameters of the fitted standard curve equations are shown in Table 2.

### 4.3. Fabrication of the Aerogel

The preparation of CaCO_3_@STA/PAM/TOCN hydrogels was carried out with reference to the literature [24]. The steps were as follows: 0.5 g of tapioca starch (adiabatic) was weighed, added to a certain volume of distilled water, and pasted in a water bath at 75 °C for 45 min. Then weigh 0.5 g of TEMPO-oxidized fiber (adiabatic) in a certain volume of distilled water and sonicate for 45 min to obtain TOCN, and mix the two solutions.

A certain amount of well-dispersed calcium carbonate nanosuspension (4% relative to the total mass of starch and nanofibers) was added. Then 4 g of acrylamide, 0.06 g of N, N′-methylenebisacrylamide, and 0.03 g of ammonium persulfate were added to the mixture. The mixture was made up to 100 g with distilled water and stirred and mixed for 10 min, after which the samples were transferred to a probe-type ultrasonic cell disintegrator. The probe was first placed in the mixture and sonicated for 10 min to mix the components uniformly.

Before the formation of the gel, the probe was removed and then sonicated in water for 45 min to obtain a crude gel product with a gel mass fraction of about 5%. During the sonication process, attention was paid to the water temperature to prevent excessive pasteification of tapioca starch due to high temperature. The obtained crude gel product was soaked in a prepared ethanol solution (80% *v*/*v*) for 24 h to obtain a hydrogel.

The obtained hydrogel samples were washed repeatedly using distilled water at 20 °C, frozen at −20 °C for 24 h, and then dried in a freeze dryer at −40 °C to finally obtain aerogels (CaCO_3_@STA/PAM/TOCN). The specific preparation steps refer to Scheme 1.

### 4.4. Characterization of Aerogel

Before SEM testing, the powder samples directly adhered to the top of the conductive gel on the sample table and then gold sprayed to eliminate static electricity. The microscopic morphological characteristics of the dry gel sections were observed. An energy-dispersive spectrometer was used for the elemental analysis to observe the distribution of calcium elements in the aerogel skeleton. A certain mass of sample (50 mg) was weighed and placed in a sample tube, degassed at 120 °C for 6 h. The adsorption and desorption capacity of the aerogel to nitrogen was tested using a QUADRASORB-EVO gas adsorber from the USA. The porosity, average pore size, and specific surface area of the dry gels were calculated from the adsorption and desorption change curves of nitrogen gas. The samples were always examined and recorded at different temperatures using a TGA209 F1 thermogravimetric analyzer from NETZSCH, Germany. The test heating conditions were: in an N2 environment, temperature range 30~700 ℃, temperature rise rate was set to 10 ℃/min, and the gas flow rate was 20 mL/min. The crystal structure analysis of the aerogels was carried out on a multifunctional horizontal X-ray diffractometer of the Nippon Rigaku Ultima IV combination type. Test conditions: X-ray tube with CuKα target (λ = 0.15406 nm), graphite monochromator to eliminate CuKα radiation, tube voltage 40 kV, tube current 200 mA, scanning range 2θ = 5~80°, scanning step 0.02°, scanning rate 15 °/min, recording “diffraction intensity-2θ” curve. The samples were determined by the KBr compression method on a German VERTEX 80 V infrared spectrometer in the wavelength range of 4000–500 cm^−1^.

### 4.5. Adsorption Kinetics

To investigate the adsorption performance of the CaCO_3_@STA/PAM/TOCN adsorbent, a single variable method was adopted for different pH values and adsorbent dosages.

For the adsorption performance under different pH conditions, 0.1 mol/L HCl and 0.1 mol/L NaOH were used to adjust the pH in the experiment. Then, 20 mg of adsorbent, CR, and MB solutions with a concentration of 100 mg/L and pH values of 2, 4, 6, 8, 10, and 12, respectively, were taken in a conical flask and the adsorption temperature was 25 ℃. The adsorption experiments were performed dynamically in a thermostatic oscillator rotational speed of 100 r/min, Guohua THZ-82, Changzhou, China. After adsorption, the supernatant was filtered with a 0.22 μm filter membrane, and the supernatant was tested for absorbance by UV spectrophotometer, choosing proper concentrations for UV analysis.

Then, the absorbance after adsorption was measured and the corresponding adsorption concentration was calculated from the standard curve. The amount of dye adsorbed and the removal rates were calculated according to the following Equations (9)–(11):

(9) $$Q_{e}=\left(C_{0}-C_{e}\right)V/m$$

(10) $$Q_{t}=\left(C_{0}-C_{t}\right)V/m$$

(11) $$\mathrm{Adsorption}\;\mathrm{efficiency}\;=\frac{\left(C_{0}-C_{e}\right)}{C_{0}}\times 100\%$$

In the equation, Q_t_, Q_e_—the amount of dye adsorbed in solution at any time and at equilibrium time, mg/g.

C_0_, C_t_, and C_e_—initial concentration of organic dyes, after adsorption and at equilibrium, mg/L.

V—the volume of dye to be adsorbed, L; m—the mass of adsorbent, g.

## Figures and Tables

**Table 1:** Comparison of the adsorption effect of the present work with other types of aerogel adsorbents.

Adsorbents	Dyes	Adsorption Capacity (mg/g)	Reference
Fe_3_O_4_@polydopamine/carboxymethyl chitosan aerogel	MB	217.4	[43]
	CV	262.3	
	MO	83.5	
	CR	92.8	
Cellulose/chitosan aerogel	CR	380.2	[44]
Graphene-cellulose-polyethyleneimine aerogel	Amaranth	369.4	[45]
	MB	237.3	
Cellulose nanofibrils aerogel	MG	203.7	[46]
Poly(propylene glycol adipate)-modified cellulose aerogel	CR	120	[47]
Cellulose nanofibrils and (3-mercaptopropyl) trimethoxysilane compressible aerogels	MO	186.7	[48]
Chitosan and cellulose nanofibrils aerogel	AB93	1428.7	[49]
Carboxymethyl cellulose-supported graphene oxide aerogel	rhodamine B	312.5	[50]
3D graphene oxide/*ι*-carrageenan composite aerogel	MB	245.3	[51]
Cationic cellulose nanofibrils aerogel	Blue dye CR19	230	[52]
	Red dye 180	160	
	Orange dye 142	560	
Chitin and psyllium biopolymers aerogel	CV	227.1	[53]
Carboxymethyl cellulose and graphene oxide composite aerogel	MB	245.0	[54]
Pineapple leaf fibers and cotton waste cellulose aerogel	MB	34.0 g/g	[55]
Silica aerogel/polyacrylonitrile/polyvinylidene fluoride nanofiber	BR18	182	[56]
Poly(methacrylic acid-co-2-(dimethylamino) ethyl methacrylate) and carboxylated cellulose nanofibrils aerogel	MB	598.8	[57]
	NGB	621.1	
	MO	892.9	
Sodium alginate/cellulose nanofibers/polyethyleneimine composite aerogel	CR	2007.5	[58]
	MO	2253.4	
Polydopamine-Modified Cellulose Nanofibril Composite Aerogel	MB	208	[59]
3D rGO/ZIF-67 aerogel	CV	1714.2	[60]
CaCO_3_@STA/PAM/TOCN aerogel	CR	277.76	This work
	MB	101.01	

Note: CR: Congo red; MG: malachite green; AB93: acid Blue 93; CV: crystal violet; MO: methyl orange; NGB: naphthol green B; BR18: basic red 18.

**Table 2:** Basic parameters and fitting standard curve equation of two organic dyes.

Dyes	Congo Red	Methylene Blue
Molecular formula	C_32_H_22_N_6_Na_2_O_6_S_2_	CHN_3_ClS
Molecular weight	696.68 g/mol	319.85 g/mol
Structural	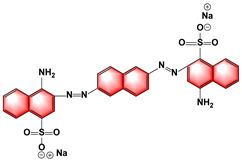	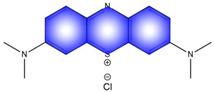
Type of dye	Anionic dye	Cationic dye
Maximum absorption wavelength/nm	499	664
Standard curve	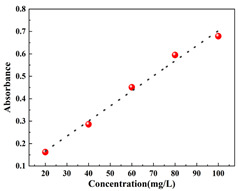	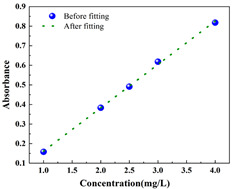
Standard curve equation	*y* = 0.0067 *x* + 0.0317	*y* = 0.2215 *x* − 0.0602
Correlation coefficient (R^2^)	0.987	0.998

Note: x: dye concentration y: absorbance corresponding to different dye concentrations.

## Data Availability

Data sharing is not applicable to this article as no datasets were generated or analyzed during the current study.
